# Omega-6:Omega-3 Fatty Acid Ratio and Total Fat Content of the Maternal Diet Alter Offspring Growth and Fat Deposition in the Rat

**DOI:** 10.3390/nu12092505

**Published:** 2020-08-19

**Authors:** Sally A. V. Draycott, Matthew J. Elmes, Beverly S. Muhlhausler, Simon Langley-Evans

**Affiliations:** 1 Sutton Bonington Campus, School of Biosciences, University of Nottingham, Loughborough LE12 5RD, UK; matthew.elmes@nottingham.ac.uk (M.J.E.); simon.langley-evans@nottingham.ac.uk (S.L.-E.); 2 Food and Nutrition Research Group, Department of Food and Wine Science, School of Agriculture, Food and Wine, University of Adelaide, Adelaide, SA 5064, Australia; beverly.muhlhausler@adelaide.edu.au; 3 Commonwealth Scientific and Industrial Research Organisation, Adelaide, SA 5000, Australia

**Keywords:** maternal nutrition, omega-6, omega-3, pregnancy, obesity, fatty acids, lipogenesis

## Abstract

Omega-3 long-chain polyunsaturated fatty acids (LCPUFA) have been shown to inhibit lipogenesis and adipogenesis in adult rats. Their possible early life effects on offspring fat deposition, however, remain to be established. To investigate this, female Wistar rats (*n* = 6–9 per group) were fed either a 9:1 ratio of linoleic acid (LA) to alpha-linolenic acid (ALA) or a lower 1:1.5 ratio during pregnancy and lactation. Each ratio was fed at two total fat levels (18% vs. 36% fat *w/w*) and offspring were weaned onto standard laboratory chow. Offspring exposed to a 36% fat diet, irrespective of maternal dietary LA:ALA ratio, were lighter (male, 27 g lighter; female 19 g lighter; *p* < 0.0001) than those exposed to an 18% fat diet between 3 and 8 weeks of age. Offspring exposed to a low LA (18% fat) diet had higher proportions of circulating omega-3 LCPUFA and increased gonadal fat mass at 4 weeks of age (*p* < 0.05). Reduced Srebf1 mRNA expression of hepatic (*p* < 0.01), gonadal fat (*p* < 0.05) and retroperitoneal fat (*p* < 0.05) tissue was observed at 4 weeks of age in male and female offspring exposed to a 36% fat diet, and hepatic Srebf1 mRNA was also reduced in male offspring at 8 weeks of age (*p* < 0.05). Thus, while offspring fat deposition appeared to be sensitive to both maternal dietary LA:ALA ratio and total fat content, offspring growth and lipogenic capacity of tissues appeared to be more sensitive to maternal dietary fat content.

## 1. Introduction

Risk of obesity may be partially attributed to the nutritional environment encountered during early life [1]. Interventions that target these critical life stages exert a greater preventative effect than those applied later in life [2]. Epidemiological as well as experimental animal studies have shown that exposure to a hypercaloric or high-fat diet during early development is associated with increased adiposity in the offspring in later life [3,4,5]. Emerging evidence, however, suggests that the type of fat an individual is exposed to during development may also play a key role in determining their future metabolic health. Of increasing interest, due to the significant increase in their consumption over the past 60 years, is the role of dietary omega-6 polyunsaturated fatty acids (PUFA) [6,7].

Knowledge of the biological effects of omega-6 fatty acids (which have pro-adipogenic and pro-inflammatory properties), as well as the evidence suggesting that increased maternal omega-6 PUFA intake is associated with offspring adiposity [8,9], has led to the hypothesis that a diet high in omega-6 PUFA may be contributing to the increased incidence of obesity [10]. Furthermore, due to the effects of the omega-3 PUFA alpha-linolenic acid (ALA) and its derivatives, which are primarily anti-inflammatory in nature, it has been hypothesised that a diet high in these fatty acids may reduce fat deposition [11,12]. Substantial increases in population level intakes of omega-6 PUFA, in particular linoleic acid (LA; precursor to longer omega-6 derivatives), have not coincided with any increases in omega-3 consumption [7], resulting in a significant increase in the ratio of omega-6 to omega-3 fatty acids in typical Western diets over the last forty years. Formation of longer-chain PUFA, such as arachidonic acid (AA; omega-6), eicosapentaenoic acid (EPA; omega-3) and docosahexaenoic acid (DHA; omega-3), relies on a common set of enzymes utilised by both families of PUFA. As such, competition exists between the two families such that the levels of omega-6 PUFA within the body can directly affect the levels of omega-3 PUFA, therefore, implying that alterations in the ratio of these two families of PUFA, as well as their overall amount, may impact on fat deposition and lipogenesis.

The potential mechanism through which variation in the omega-6:omega-3 ratio in early life may programme long-term metabolic health is unknown. It is possible that early changes in the patterns of expression of key genes involved in lipogenesis within the liver and adipose tissue have a long-term impact on fat deposition and accumulation. These genes include sterol regulatory element-binding protein 1c (*Srebf1*), peroxisome proliferator-activated receptor gamma (*Pparg*), fatty acid synthase (*Fasn*), lipoprotein lipase (*Lpl*), and leptin (*Lep*). Previous studies in adult animals have also demonstrated that increased omega-3 PUFA intake can reduce lipid accumulation resulting in an overall reduction in body fat [11,12,13], and that this is mediated through modulation of the expression of *Srebf1* [14] and *Pparg* [15,16]. There have been few studies, however, investigating whether these anti-lipogenic effects are observed in offspring exposed to a maternal diet that is high in omega-3 fats. Conflicting results have been reported in this regard, with some studies reporting decreased [9,17,18] and others reporting increased [19] offspring adiposity.

The aim of this study was to investigate the effects of feeding a maternal dietary LA:ALA ratio similar to that of the Western diet (9:1) [7], compared to a proposed ‘ideal’ ratio of~1:1.5 [20,21] on offspring adiposity and other health indicators in rats. To elucidate any additive effects of altering the maternal dietary LA:ALA ratio, each diet was fed at either 18% fat *w/w* or at a higher fat content of 36% fat *w/w*. This paper focusses specifically on the effects of pre- and early postnatal exposure to altered dietary fat content and fatty acid ratio on offspring that have been weaned onto a standard laboratory diet. As such, offspring are no longer directly exposed to the maternal dietary intervention postweaning. We hypothesised that exposure to a high LA diet during pregnancy and lactation would lead to increased adiposity in the offspring, in conjunction with an increased expression of lipogenic genes, and that this effect may be exacerbated with exposure to a high-fat diet.

## 2. Materials and Methods

### 2.1. Animals

All animal procedures were performed in accordance with the Animals (Scientific Procedures) Act 1986 under Home Office licence and were approved by the Animal Ethics Committee of the University of Nottingham, UK (Project code 40/3598; approved 02/03/2015). Virgin female Wistar rats (*n* = 30; 75–100 g; Charles River, UK) were maintained as previously described [22]. After acclimatisation, a tail vein blood sample was taken from each animal for the determination of fatty acid status and individuals were then randomly allocated to experimental groups. Animals were maintained on their allocated diet for a four week ‘pre-feeding’ period, after which they were mated. Conception was confirmed by the presence of a semen plug and this was recorded as day 0 of pregnancy. Animals were housed in individual cages and remained on their respective diets throughout pregnancy and lactation. All maternal data are reported elsewhere [22].

Litters were standardised to 8 pups within 24 h of birth (4 males and 4 females, where possible). At 1 and 2 weeks of age, one male and one female from each litter were euthanised and tissues collected for analyses, the results of which are published elsewhere [22]. At 3 weeks of age, the remaining offspring were weaned and dams were euthanised by CO_2_ asphyxiation and cervical dislocation for collection of maternal blood and tissues. Offspring were weaned onto a standard laboratory chow diet (2018 Teklad Global 18% Protein Rodent Diet, Harlan Laboratories, Derby, UK) and pair-housed with the remaining same sex littermate. Offspring bodyweight was measured weekly and all animals had blood pressure measured at 4 weeks of age. At this time, one male and one female were euthanised by CO_2_ asphyxiation and cervical dislocation. Blood pressure was measured again at 8 weeks of age in all remaining animals after which the experiment ended and all remaining animals were euthanised by CO_2_ asphyxiation and cervical dislocation.

### 2.2. Diets

Diets were designed to provide either a high (9:1, high LA) or low (1:1.5, low LA) ratio of LA to ALA. For each level of LA, diets containing either 18% or 36% fat (*w/w*) were developed. This resulted in four experimental diets; high LA (18% fat; *n* = 6), high LA (36% fat; *n* = 8), low LA (18% fat; *n* = 7) and low LA (36% fat; *n* = 9). The list of ingredients and final fatty acid composition of the four experimental diets are reported elsewhere [22].

### 2.3. Tail Cuff Plethysmography

This experiment utilised a non-invasive method for measuring blood pressure validated by Feng, et al. [23]. A volume pressure recording (VPR) sensor was used to measure tail blood volume to assess systolic, diastolic and mean arterial blood pressure as well as heart rate. Prior to blood pressure measurements, animals were placed in a heat box set to 30 °C for 15 min to enhance blood flow to the tail. Animals were then restrained in individual restraint tubes with an adjustable nose cone, fitted with the deflated occlusion and VPR cuff (CODA System, Kent Scientific, Torrington, CT, USA), and left to acclimatise to the restraint tube for 10 min to minimise the impact of stress before measurements began. After acclimatisation, animals underwent 10 cycles of blood pressure measurements; of these 10 cycles, the first three were disregarded as acclimatisation cycles and an average for each measurement was taken from the remaining seven. Animals were restrained for no longer than 30 min and removed if they exhibited any signs of stress.

### 2.4. Blood Sample and Tissue Collection

Blood samples were collected from the offspring, when culled, at 4 and 8 weeks of age via cardiac puncture and ~30 µL was spotted onto PUFAcoat™ dried blood spot (DBS) collection paper (Waite Lipid Analysis Service, Adelaide, Australia [24]), allowed to dry at room temperature and stored at −20 °C for subsequent fatty acid analysis. The remainder of the blood sample was centrifuged at 13,000 rpm for 10 min at 4 °C. The plasma was isolated from the whole blood sample and stored at −80 °C until further analysis. Offspring body and organ weights were measured and samples of liver, gonadal fat and retroperitoneal fat were collected at each time point. All tissue samples were snap-frozen in liquid nitrogen and stored at −80 °C until determination of gene expression by quantitative reverse transcriptase PCR (qRT-PCR).

### 2.5. Lipid Extraction

Total lipids were extracted from liver samples of 4- and 8-week-old offspring. For each sample, ~300 mg of crushed, frozen liver was homogenised in 1.6 mL of 0.5M Na_2_SO_4_. The homogenate was decanted into 5.4 mL of hexane-isopropanol (3:2, *v/v*) and 2 mL of 0.5M Na_2_SO_4_ was added. Samples were vortexed and then centrifuged at 3000 rpm for 15 min. The supernatant was removed into a fresh tube, dried under nitrogen and the resultant lipid content was weighed. Samples were resuspended in 1 mL of hexane and 100 µL of resuspended sample was removed into a fresh tube, re-dried under nitrogen and resuspended in 100 µL of isopropanol for the determination of cholesterol and triglyceride content. The remaining sample was stored at −20 °C for fatty acid analysis.

### 2.6. Determination of Circulating and Hepatic Lipids

Plasma and liver cholesterol and triacylglycerol (TAG) content was determined by a quantitative enzymatic colorimetric assay as per the manufacturer’s protocol (Infinity™ cholesterol and Infinity™ triglyceride reagent; Thermo Scientific, Abingdon, UK).

### 2.7. Fatty Acid Methylation and Fatty Acid Analysis of Whole Blood and Liver Samples

Fatty acid composition in maternal and foetal whole blood, and in lipids extracted from liver samples from offspring at 4 weeks of age, was determined by Gas Chromatography (GC) on a Hewlett-Packard 6890 gas chromatograph using methods that have previously been described in detail [22,24]. Individual fatty acid content was calculated based on peak area and response factors normalised to total fatty acid content and expressed as a percentage of total fatty acids.

### 2.8. Isolation of RNA and cDNA Synthesis and Reverse Transcription Quantitative Real-Time PCR (qRT-PCR)

RNA was isolated from crushed snap-frozen samples of ~25 mg of liver using the Roche High Pure Tissue kit (Roche Diagnostics Ltd., Burgess Hill, UK). Adipose RNA was extracted, after homogenisation of ~100 mg of tissue with MagNA Lyser green beads and instrument (Roche Diagnostics Ltd., Burgess Hill, UK), using the RNeasy Mini Kit (QIAGEN Ltd., Manchester, UK). RNA concentration was determined using a Nanodrop 2000 (Thermo Scientific, Abingdon, UK) and RNA quality was evaluated by agarose gel electrophoresis. cDNA was synthesised using a RevertAid™ reverse transcriptase kit (Thermo Fisher Scientific, Abingdon, UK) with random hexamer primers.

Lipogenic pathway and adipokine target genes included peroxisome proliferator-activated receptor gamma (*Pparg*), sterol regulatory element-binding protein (variant 1c; *Srebf1*), fatty acid synthase (*Fasn*), lipoprotein lipase (*Lpl*) and leptin (Lep). Primer sequences for these gene targets have previously been published elsewhere [22]. Hepatic expression of delta-5 (*Fads1*; Rn_Fads1_1_SG QuantiTect Primer Assay, Qiagen) and delta-6 (*Fads2*; Rn_Fads2_1_SG QuantiTect Primer Assay, Qiagen) desaturase enzymes were also determined. Cyclophilin A (*Ppia*) and β-actin (*Actb*) were used as housekeeper genes. Adipocyte and hepatic gene expression was quantified using SYBR Green (Roche Diagnostics) in a Light-Cycler 480 (Roche Diagnostics). Samples were analysed against a standard curve of a serially diluted cDNA pool to produce quantitative data and expression was normalised to the housekeeping gene using LightCycler^®^ 480 software (version 1.5.1) as previously described [25]. The expression of the housekeeper genes were not different between treatment groups.

### 2.9. Statistical Analysis

Data are presented as the mean ± SEM. Data were analysed using the Statistical Package for Social Sciences (Version 24, SPSS Inc., IBM, Chicago, IL, USA). The effect of maternal dietary fatty acid ratio, maternal dietary fat content and sex on dependent variables was assessed using a three-way ANOVA. Where sex had a main effect on variables but no interaction with maternal dietary factors, data were split for male and female offspring and a two-way ANOVA was then used to assess the effect of maternal dietary fat content and fatty acid ratio on male and female offspring separately. Where longitudinal data were analysed, as with bodyweight, the impact of maternal dietary LA:ALA ratio and maternal dietary fat content was analysed using a two-way repeated-measures ANOVA. A value of *p* < 0.05 was considered to be statistically significant and dams were used as the unit of analysis.

## 3. Results

### 3.1. Offspring Bodyweight, Body Composition and Blood Pressure

**Figure 1:**
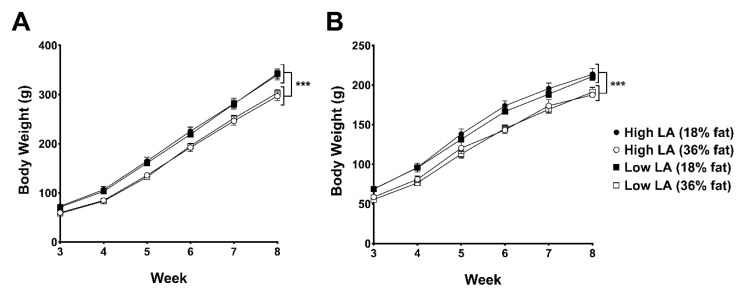
Body weights of (**A**) male and (**B**) female offspring postweaning up to 8 weeks of age exposed to either a high LA (18% fat) diet (closed circles), high LA (36% fat) diet (open circles), low LA (18% fat) diet (closed squares) or a low LA (36% fat) diet (open squares) during gestation and lactation. Offspring were weaned onto a chow diet. Values are means ± SEM and *n* = 6–9 per group. The effects of dietary fatty acid ratio and dietary fat content were determined using a two-way repeated measures ANOVA. *** indicates a significant effect of maternal dietary fat content (*p* < 0.0001) on body weight.

Figure 1 shows bodyweights of offspring from 3 to 8 weeks of age. Offspring birthweight and bodyweight prior to this are reported elsewhere [22]. Offspring of dams consuming a 36% fat diet, irrespective of maternal dietary LA:ALA ratio, were lighter than offspring of dams fed on an 18% fat diet from 3 to 8 weeks of age in both male (on average 27 g lighter) and female (on average 19 g lighter) offspring (*p* < 0.0001).

Table 1 shows the organ and fat depot weights of male and female offspring normalised to bodyweight at 4 and 8 weeks of age (absolute organ weights can be found in Supplementary Table S1). At 4 weeks of age, relative heart weight was 5% higher and relative liver weight was 4% lower in female offspring of dams exposed to a 36% fat diet compared to those exposed to an 18% fat diet, irrespective of maternal dietary LA:ALA ratio (*p* < 0.05). Relative liver weight at 4 weeks also tended (*p* = 0.075) to be lower in male offspring of dams consuming the 36% vs. 18% fat diet. A significant (*p* < 0.05) interaction between maternal dietary fatty acid ratio and maternal dietary fat content on relative gonadal fat weight was observed for both male and female offspring at 4 weeks of age. This manifested as ~30% lower weight of the gonadal fat depots in the low LA group, but only if exposed to a 36% fat diet in early life. There were no differences in the relative weight of lungs, kidneys or retroperitoneal fat pads between experimental groups at 4 weeks of age. Differences in relative organ and fat weights measured in offspring at 4 weeks of age appeared to be transient, as no differences were observed at 8 weeks of age for any of these organs or fat depots.

Blood pressure at 4 weeks of age was not influenced by maternal diet. At 8 weeks of age, female offspring exposed to a 36% diet during gestation and lactation had significantly lower systolic (16 mmHg; *p* = 0.024) and tended to have lower diastolic (11 mmHg; *p* = 0.068) blood pressure than offspring exposed to an 18% fat diet (Table 1). Blood pressure in males was not influenced by either LA:ALA ratio or fat content of the maternal diet.

### 3.2. Offspring Whole Blood and Hepatic Fatty Acid Profile

**Figure 2:**
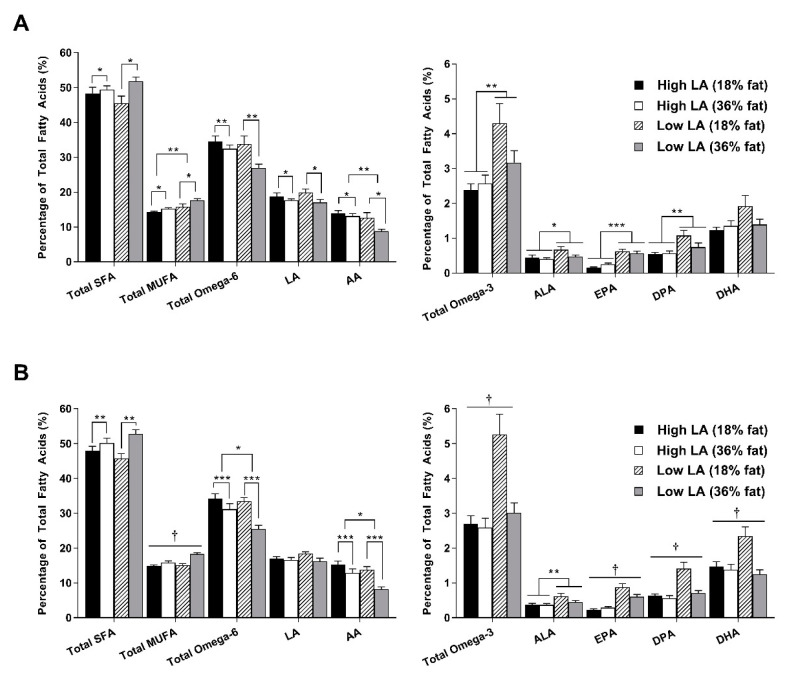
Whole blood fatty acid profile in (**A**) male and (**B**) female offspring at 4 weeks of age. Values are means ± SEM and *n* = 6–9 per group. The effects of maternal dietary fatty acid ratio and maternal dietary fat content were determined using a two-way ANOVA; all comparisons were made within sex group. * Indicates significant difference (* *p* < 0.05, ** *p* < 0.01, *** *p* < 0.001). † indicates a significant interaction effect (*p* < 0.05). SFA, saturated fatty acid; MUFA, monounsaturated fatty acid; LA, linoleic acid; AA, arachidonic acid; ALA, alpha-linolenic acid; EPA, eicosapentaenoic acid; DPA, docosapentaenoic acid; DHA, docosahexaenoic acid.

A significant effect of sex was observed for some of the fatty acids measured in whole blood and liver at 4 and 8 weeks of age. However, no interactions were observed between sex and maternal dietary treatment, so male and female data were split for further analysis. Figure 2 shows the fatty acid profile of whole blood in offspring at 4 weeks of age. In male offspring, exposure to a 36% fat diet was associated with increased proportions of saturated fatty acids (SFA; *p* < 0.05) and monounsaturated fatty acid (MUFA; *p* < 0.05) as well as decreased proportions of LA (*p* < 0.05) and AA (*p* < 0.05), resulting in lower overall total omega-6 in response to a maternal 36% fat diet. Proportions of MUFA and AA were also influenced by maternal dietary fatty acid ratio such that a low LA diet was associated with increased MUFA (*p* < 0.01) and decreased AA (*p* < 0.01) levels. A similar pattern was observed for the proportions of SFA, MUFA and omega-6 fatty acids in whole blood of female offspring. In females, a significant interaction was observed for the proportions of long-chain omega-3 fatty acids (EPA, *p* < 0.05; DPA, *p* < 0.01 and DHA; *p* < 0.05). Interestingly, female offspring exposed to a low LA (18% fat) diet had higher proportions of these fatty acids and as a result, higher total omega-3 proportions. Similar patterns were observed in male offspring. However, only a significant main effect of maternal dietary LA:ALA ratio was observed.

**Figure 3:**
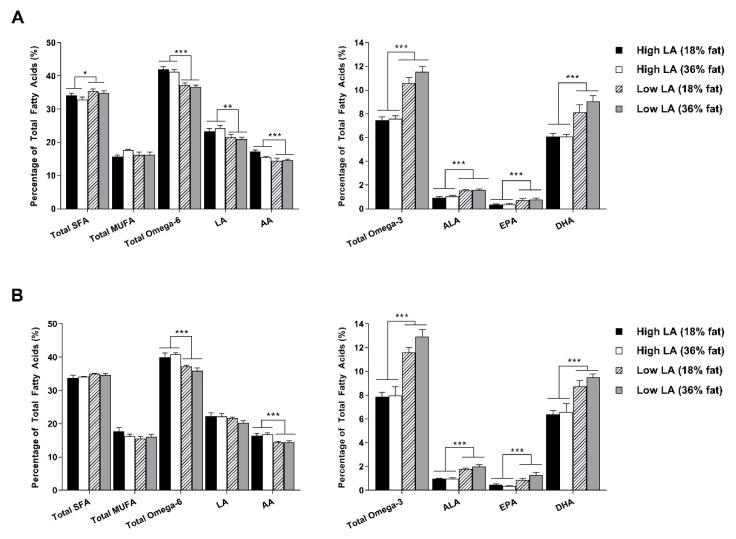
Liver fatty acid profile in (**A**) male and (**B**) female offspring at 4 weeks of age. Values are means ± SEM and *n* = 6–9 per group. The effects of maternal dietary fatty acid ratio and maternal dietary fat content were determined using a two-way ANOVA; all comparisons were made within sex group. * Indicates significant difference (* *p* < 0.05, ** *p* < 0.01, *** *p* < 0.001). SFA, saturated fatty acid; MUFA, monounsaturated fatty acid; LA, linoleic acid; AA, arachidonic acid; ALA, alpha-linolenic acid; EPA, eicosapentaenoic acid; DPA, docosapentaenoic acid; DHA, docosahexaenoic acid.

The elevated omega-3 proportions in offspring of dams exposed to a low LA (18% fat) diet at 4 weeks of age, prompted investigation into the liver fatty acid profile at this time point (Figure 3). Interestingly, the composition of fatty acids in the liver did not completely reflect that of the whole blood and were only influenced by maternal dietary fatty acid ratio. In male offspring, exposure to a low LA diet during pregnancy and lactation was associated with lower proportions of total omega-6, LA and AA and higher proportions of total omega-3, ALA, EPA, DHA and total SFA in the liver (Figure 3A). Similar observations were made for the fatty acid composition of the liver in female offspring at this time point. A key difference, however, was that maternal diet appeared to have no effect on total SFA in the female offspring (Figure 3B).

**Figure 4:**
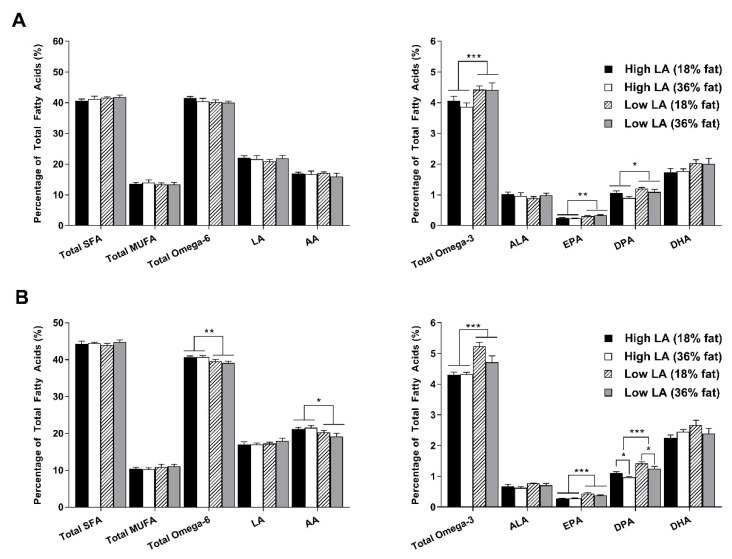
Offspring whole blood fatty acid profile in (**A**) male and (**B**) female offspring at 8 weeks of age. Values are means ± SEM and *n* = 6–9 per group. The effects of maternal dietary fatty acid ratio and maternal dietary fat content were determined using a two-way ANOVA. All comparisons were made within sex group. * Indicates significant difference (* *p* < 0.05, ** *p* < 0.01, *** *p* < 0.001). SFA, saturated fatty acid; MUFA, monounsaturated fatty acid; LA, linoleic acid; AA, arachidonic acid; ALA, alpha-linolenic acid; EPA, eicosapentaenoic acid; DPA, docosapentaenoic acid; DHA, docosahexaenoic acid.

At 8 weeks of age, whole blood fatty acid profile was reassessed (Figure 4). In male offspring, there were no longer any differences in proportions of SFA, MUFA, total omega-6, LA, AA or ALA between experimental groups. Total omega-3 (*p* < 0.001), EPA (*p* < 0.01), DPA (*p* < 0.05) and DHA (*p* = 0.052) proportions all remained elevated in male offspring of dams exposed to a low LA diet during pregnancy and lactation. Similar observations were made for the fatty acid composition of female whole blood at 8 weeks of age. Proportions of AA, and consequently levels of total omega-6, were, however, higher in female offspring exposed to a high LA diet, irrespective of maternal dietary fat content. In both male and female whole blood, DPA proportions appeared to be influenced by maternal dietary fat content such that a 36% fat diet was associated with lower proportions of this fatty acid. This was significant in female offspring (*p* < 0.05) and tended towards significance in male offspring (*p* = 0.057). Unlike in male offspring, DHA proportions in female offspring whole blood at 8 weeks of age were not associated with maternal dietary intake.

### 3.3. Circulating and Hepatic Lipid Profile

At 4 weeks of age, male offspring exposed to a 36% fat diet had lower circulating plasma TAG concentrations (*p* = 0.01) and reduced liver cholesterol concentrations (*p* < 0.05) when compared to offspring exposed to an 18% fat diet (Table 2). In the female liver, however, TAG concentrations were affected by maternal dietary ratio such that female offspring exposed to a low LA diet had lower concentrations of liver TAG (*p* < 0.05), irrespective of maternal dietary fat level. There was no effect of maternal diet on plasma cholesterol or total liver lipid at 4 weeks of age. By 8 weeks of age, there were no significant differences in any of the variables measured in female offspring. In males, however, there was a significant interaction of maternal dietary fat content and fatty acid ratio such that exposure to a high LA (36% fat) diet resulted in increased circulating cholesterol (*p* < 0.05) but reduced liver TAG concentrations in 8-week-old male offspring (Table 2).

### 3.4. Gene Expression

An interaction between maternal dietary fatty acid ratio and fat content on hepatic expression of *Fads1* and *Fads2* was observed in female offspring (Table 2). This resulted in increased expression of both genes in female offspring of dams exposed to a high LA (36% fat) diet suggesting an increased capacity for synthesis of long-chain fatty acids in this group. There were no differences in the expression of these genes in male offspring. Expression of key lipogenic genes (*Fasn*, *Lpl*, *Pparg*, *Srebf1* and *Lep*) was measured in the liver as well as gonadal and retroperitoneal fat depots (*Lep* was only measured in the fat depots due to limited hepatic expression). At 4 weeks of age, a consistent effect of maternal dietary fat content on expression of *Srebf1* mRNA was observed (Table 3). Offspring of dams exposed to a 36% fat diet had lower expression of hepatic (*p* < 0.01), gonadal fat (*p* < 0.05; significant in female offspring only) and retroperitoneal fat (*p* < 0.05) *Srebf1* mRNA compared to offspring of dams consuming an 18% fat diet. A similar pattern was observed for other genes in the retroperitoneal fat depot, such that offspring of dams exposed to a 36% fat diet exhibited lower mRNA expression of *Fasn* (*p* < 0.01; male offspring only), *Lpl* (*p* < 0.05; male and female offspring) and *Lep* (*p* < 0.05; male offspring only). At 4 weeks of age, hepatic *Lpl* expression was higher in offspring of dams consuming a low LA 36% fat diet compared to other groups in both male and female offspring (*p* < 0.05). In female offspring at 4 weeks of age, a significant interaction between maternal dietary fatty acid ratio and maternal dietary fat content was observed in gonadal fat expression of *Fasn* (*p* < 0.05) and *Lep* (*p* < 0.05) as well as retroperitoneal fat expression of *Pparg* (*p* < 0.05) and *Lep* (*p* < 0.01). This interaction manifested as increased expression of these genes in offspring exposed to 36% fat with a high LA:ALA ratio, but decreased expression when the diet consisted of a low LA:ALA ratio. As such, offspring exposed to a low LA (18% fat) diet consistently exhibited the highest expression of these genes.

Table 4 summarises the mRNA expression at 8 weeks of age. Male offspring exposed to a 36% fat diet during gestation and lactation showed lower hepatic *Srebf1* and gonadal fat *Lep* mRNA expression (*p* < 0.05) when compared to an 18% fat diet, irrespective of maternal dietary fatty acid ratio. In females, and other tissues measured in male offspring, the differences in *Srebf1* expression observed at 4 weeks of age appeared to be transient, as no differences were observed between groups at 8 weeks of age. *Fasn* mRNA at 8 weeks of age was significantly higher in female offspring of dams consuming a 36% fat diet in both the gonadal (*p* < 0.05) and retroperitoneal (*p* < 0.01) fat depots, irrespective of maternal dietary ratio. 

## 4. Discussion

This study aimed to investigate the effect of an altered maternal dietary LA:ALA ratio, as well as total dietary fat content, on offspring growth, adiposity, lipid profiles and expression of key genes associated with lipogenesis. We have shown that the maternal dietary LA:ALA ratio is a key driver of the fatty acid profile in whole blood and liver of adult offspring. Additionally, we found that exposure to a high-fat diet, irrespective of dietary LA:ALA ratio, was associated with a reduction in offspring bodyweight that persisted after the offspring were weaned onto a standard, nutritionally balanced rodent diet. Differences in adipose tissue weight were determined by maternal dietary LA:ALA ratio as well as total fat content, whilst the expression of key lipogenic genes was predominantly affected by the latter. These data suggest that a maternal diet high in fat can have detrimental effects on offspring growth whilst an interaction between total fat intake and maternal dietary PUFA ratio appears to affect offspring adiposity via alterations in the expression of lipogenic genes.

We have previously shown that exposure to a varying LA:ALA ratio and fat content in the diet influences the circulating fatty acid profile of dams [22] as well as offspring directly exposed to the maternal diet [26]. In the present study, we have demonstrated that the circulating and hepatic fatty acid profiles of offspring at 4 weeks of age, as well as the circulating fatty acid profile at 8 weeks of age, are still influenced by maternal dietary factors despite the offspring no longer being directly exposed to dietary interventions. Of particular interest is the elevated proportions of long-chain omega-3 PUFA in whole blood samples of offspring exposed to a low LA (18% fat) diet but not in those exposed to a low LA (36% fat) diet. The experimental diet, as well as the chow diet that offspring were weaned onto, only contained the omega-6 and omega-3 precursors, LA and ALA. This implies, therefore, that the increased levels of long-chain omega-3 PUFA (LCPUFA) are due to an increased capacity within these offspring to convert ALA to its longer-chain derivatives through elongation and desaturation and/or remnants of preferential transfer of these fatty acids from the mother during pregnancy and/or lactation. We are inclined to believe this is a result of the latter as our previous study indicated a similar fatty acid profile in the dams during the lactation period [26]. Further to this, studies in other species have provided no evidence of increased desaturation and elongation capacity of offspring exposed to higher omega-3 levels [27]. However, a combination of these factors, as well as the possible influence of fatty acid release from adipose tissue, is conceivable and should not be completely ruled out.

This interesting finding in the offspring whole blood fatty acid profile at 4 weeks of age encouraged investigation into the hepatic fatty acid profile and capacity for long-chain PUFA synthesis. Interestingly, and despite evidence of strong correlations between circulating and hepatic liver fatty acid profiles [28], we found that the increased omega-3 LCPUFA observed in whole blood of offspring exposed to a low LA (18% fat) diet was not apparent in the liver. Investigation into the desaturation capacity of the liver in these animals revealed some sex-specific interactions of maternal diet and key genes associated with this pathway. The observation that female offspring exposed to a high LA (36% fat) diet exhibited increased levels of *Fads1* and *Fads2*, does in fact suggest that these individuals may have an increased capacity for LCPUFA synthesis. This did not, however, appear to translate into any physiological differences in the composition of fatty acids in the liver between experimental groups and the mRNA levels measured in this study may not be reflective of protein levels and/or activity of these enzymes. In addition, assessments of enzyme activity and mRNA expression of elongase enzymes would provide further insights into the capacity for LCPUFA synthesis in the liver. These findings do, however, highlight the potential for prolonged biological effects of fatty acids incorporated into phospholipid membranes and/or stored in tissues during gestation and lactation. Further experiments investigating the longevity of changes in offspring fatty acid profiles would confirm if there is a programmed effect of increased capacity for LCPUFA synthesis or if this is an artefact of direct exposure to the maternal dietary intervention. Even if transient, the effects of altered fatty acid composition of tissues, restriction of growth and greater adiposity that we have observed are likely to potentiate long-term metabolic consequences.

Offspring of dams exposed to a 36% fat diet exhibited consistently lower bodyweights than offspring exposed to an 18% fat diet during gestation and lactation. Importantly, this effect was apparent from birth [26] and persisted after the offspring had been weaned onto a standard laboratory diet, suggesting a long-term effect of exposure to a maternal high-fat diet that is persistent beyond direct dietary exposure. This is consistent with many studies reporting decreased foetal [29,30], birth [31] and weaning weight [32] in offspring of dams exposed to a high-fat diet during gestation and lactation periods. Early life growth restriction is often proceeded by a period of “catch-up” growth in which offspring gain weight rapidly and is often associated with increased adiposity [33] and increased risk of metabolic disease and hypertension in the offspring [34]. One possibility is that the decreased bodyweight observed in this group was due to reduced feed intake, although we were not able to assess this in the current study as animals were group housed. Previous studies have, however, reported alterations in the energy intake and neuroendocrine control of bodyweight in offspring of dams exposed to a high-fat diet [35,36] adding feasibility to this hypothesis, and it would be interesting to assess these parameters in future studies using similar diets to the current study. Surprisingly, further to a reduced bodyweight in response to a maternal high-fat diet, female offspring at 8 weeks of age also had reduced blood pressure. This apparent increased sensitivity of female rather than male offspring to maternal dietary treatments has been noted previously [37], although, as in a number of other studies [38,39], maternal high-fat diets resulted in increased as opposed to decreased blood pressure. It is important to note, however, that many of these studies utilised a maternal diet high in saturated fat and studies using diets high in polyunsaturated fats, as with this study, have shown reductions in blood pressure when compared to a maternal diet high in saturated fats [40]. In support of this, we have previously noted that offspring hypertension associated with low protein feeding during rat pregnancy is modified by other components of the experimental diet, including the source of fat [41,42].

An unexpected finding of this study was that offspring exposed to a low LA (18% fat) diet had the highest relative gonadal fat mass at 4 weeks of age, in conjunction with higher *Lep* mRNA expression in the gonadal fat adipose tissue. This was consistent across sexes and conflicted with our hypothesis as well as the evidence linking increased omega-3 intake with reduced fat deposition and accumulation in in vitro and rodent models [9,18]. This finding was, however, in line with other rodent studies in which the higher omega-3 exposure was restricted to the gestation and lactation periods [19] and adds to the disparity observed in reports of human [43] as well as animal studies [44] investigating the role of increased maternal dietary omega-3 on offspring body composition. The increased gonadal fat weight of the low LA (18% fat) group at 4 weeks of age coincided with increased *Fasn* expression in this tissue, suggesting that the higher gonadal fat deposition may have been driven by an increased capacity for de novo lipogenesis in this group. Interestingly, however, this increased *Fasn* expression was only observed in females, raising the possibility of different underlying mechanisms for increased gonadal fat accumulation between males and females which has also been suggested by previous work [45].

Interestingly, and despite no effects on relative fat mass, expression of lipogenic genes in the retroperitoneal fat depot appeared to be more susceptible to maternal dietary effects compared to the gonadal fat depot. In both male and female offspring at 4 weeks of age, there appeared to be a decrease in lipogenic capacity in offspring of dams fed high-fat diets. This apparent reduction in lipogenic capacity may be a compensatory response to mitigate the effects of a maternal high-fat diet and limit excessive fat accumulation in these groups which has been demonstrated in rodent models directly consuming a high-fat diet [46,47,48,49]. Alternatively, dietary PUFA can act as potent inhibitors of lipogenesis [50]. It may be that the high amount of PUFA that dams are consuming as part of the high-fat diets within this study was sufficient to reduce expression of lipogenic genes in the offspring. To our knowledge, few studies have investigated the effect of maternal dietary PUFA on offspring lipogenic capacity, and one such study found no effect of maternal high omega-3 diet on the expression of key lipogenic genes in the offspring [19]. Therefore, further studies are required to more fully understand the impact of maternal fat intake on lipogenesis in the offspring. Indications of reduced lipogenic capacity were also apparent in the liver in offspring of dams exposed to a high-fat diet. A significant reduction in hepatic *Srebf1* expression was observed in the offspring of dams receiving a 36% fat diet; this was accompanied by proportionally lower liver weights in these groups at 4 weeks of age. In other models of maternal dietary insult, such as the low protein model, studies have shown that early reductions in the lipogenic capacity of tissues, through reduced gene expression, are often followed by an upregulation in lipogenesis between 9 and 18 months of age [51], but can occur much earlier if the individual encounters further dietary challenge [52]. Whilst this study only followed offspring until 8 weeks of age, some indications of this shift in lipogenic capacity were apparent in female offspring at this time point. Female offspring of dams exposed to a 36% fat diet exhibited increased *Fasn* levels suggesting increased de novo lipogenesis in these tissues. It is important to note, however, that although the genes investigated within this study are key regulators of lipogenesis, the list is not exhaustive, and inclusion of additional lipogenic genes, as well as genes involved in inflammatory pathways, would provide additional insights. Further to this, it will be of interest in future studies to determine whether the observed changes in phenotype and/or gene expression persist or change as the offspring age.

In conclusion, we have shown that, despite significant alteration in the ratio of omega-3 and omega-6 fatty acids in offspring of dams fed either a high or low LA diet, offspring growth and lipogenic capacity of adipose tissue are more susceptible to changes in the total fat content of the maternal diet rather than changes in the types of fats consumed. Whilst there appears to be more robust data supporting the beneficial effects of omega-3 fatty acids on mature adipocytes [11,12,13], their biological effects on developing adipose tissue are far less clear. Evidence suggesting beneficial or detrimental effects of the two families of PUFA in the maternal diet on offspring growth and adiposity, have largely been based on in vitro studies or animal experiments and recent data have suggested limited reproduction of these results in human trials [53]. Further studies are required to investigate the effects of maternal dietary PUFA on developing tissues but caution should be exercised in the meantime not to extrapolate from data on mature tissues and to highlight the detrimental effects of a maternal high-fat intake regardless of the types of fats consumed.

## Figures and Tables

**Table 1:** Offspring body composition and blood pressure at 4 and 8 weeks of age.

Experimental Group	Male				Female			
	High LA (18% Fat)	High LA (36% Fat)	Low LA (18% Fat)	Low LA (36% Fat)	High LA (18% Fat)	High LA (36% Fat)	Low LA (18% Fat)	Low LA (36% Fat)
**4 Week Offspring**								
Heart (% BW)	0.49 ± 0.02	0.53 ± 0.02	0.50 ± 0.02	0.51 ± 0.02	0.52 ± 0.003 ^a^	0.55 ± 0.002 ^b^	0.51 ± 0.02 ^a^	0.53 ± 0.01 ^b^
Lungs (% BW)	1.05 ± 0.07	1.07 ± 0.10	1.02 ± 0.07	1.29 ± 0.06	1.39 ± 0.09	1.28 ± 0.12	1.20 ± 0.11	1.19 ± 0.08
Kidney (%BW)	1.08 ± 0.03	1.12 ± 0.03	1.10 ± 0.02	1.06 ± 0.01	1.02 ± 0.10	1.11 ± 0.02	1.13 ± 0.04	1.05± 0.02
Liver (% BW)	5.05 ± 0.15	4.92 ± 0.13	5.05 ± 0.12	4.71 ± 0.10	4.98 ± 0.07 ^a^	4.73 ± 0.08 ^b^	4.76 ± 0.16 ^a^	4.60 ± 0.11 ^b^
Gonadal fat (%BW) *	0.49 ± 0.03	0.52 ± 0.04	0.56 ± 0.03	0.39 ± 0.03	0.64 ± 0.09	0.66 ± 0.05	0.79 ± 0.05	0.54 ± 0.04
Retroperitoneal fat (%BW)	0.57 ± 0.06	0.52 ± 0.04	0.52 ± 0.07	0.47 ± 0.05	0.42 ± 0.06	0.37 ± 0.03	0.42 ± 0.03	0.34 ± 0.03
Systolic BP (mmHg)	86.2 ± 4.0	92.6 ± 4.6	89.0 ± 5.9	91.9 ± 3.8	85.8 ± 7.2	87.4 ± 3.3	91.8 ± 4.1	91.3 ± 5.2
Diastolic BP (mmHg)	65.5 ± 3.6	68.2 ± 3.7	65.4 ± 4.6	68.3 ± 3.3	61.9 ± 7.1	61.1 ± 2.1	68.9 ± 4.1	64.5 ± 2.2
**8 Week Offspring**								
Brain (%BW)	0.56 ± 0.02	0.57 ± 0.02	0.55 ± 0.02	0.57 ± 0.01	0.80 ± 0.01	0.84 ± 0.02	0.79 ± 0.01	0.79 ± 0.03
Heart (% BW)	0.34 ± 0.01	0.37 ± 0.01	0.36 ± 0.01	0.38 ± 0.01	0.39 ± 0.01	0.40 ± 0.01	0.39 ± 0.02	0.40 ± 0.01
Lungs (% BW)	0.61 ± 0.06	0.62 ± 0.05	0.61 ± 0.05	0.63 ± 0.04	0.66 ± 0.04	0.68 ± 0.04	0.60 ± 0.02	0.68 ± 0.05
Kidney (%BW)	0.83 ± 0.04	0.83 ± 0.02	0.84 ± 0.02	0.88 ± 0.03	0.88 ± 0.03	0.87 ± 0.03	0.85 ± 0.03	0.81 ± 0.02
Liver (% BW)	4.80 ± 0.08	5.00 ± 0.14	4.96 ± 0.09	5.00 ± 0.08	4.49 ± 0.16	4.63 ± 0.08	4.43 ± 0.10	4.56 ± 0.08
Gonadal fat (%BW)	1.30 ± 0.10	1.25 ± 0.08	1.34 ± 0.08	1.19 ± 0.09	1.38 ± 0.15	1.56 ± 0.07	1.53 ± 0.19	1.57 ± 0.23
Retroperitoneal fat (%BW)	1.22 ± 0.09	1.29 ± 0.11	1.32 ± 0.11	1.16 ± 0.10	0.88 ± 0.10	0.94 ± 0.11	0.94 ± 0.09	0.86 ± 0.08
Systolic BP (mmHg)	116.0 ± 5.0	109.1 ± 5.2	113.4 ± 3.4	105.8 ± 6.6	122.7 ± 10.1 ^a^	103.2 ± 3.0 ^b^	120.4 ± 8.2 ^a^	107.4 ± 5.9 ^b^
Diastolic BP (mmHg)	82.9 ± 0.2	73.5 ± 5.3	80.3 ± 3.3	74.4 ± 5.7	82.3 ± 5.1	71.8 ± 2.3	86.0 ± 7.1	74.5 ± 6.3

All values are mean ± SEM and organ weights are expressed as a percentage of bodyweight (%BW). A two-way ANOVA was used to analyse results with maternal dietary fatty acid ratio and maternal dietary fat content as factors, all comparisons are made within sex groups. Different superscripts (a, b) denote values which are significantly different (*p* < 0.05). * indicates a significant interaction effect of maternal dietary fatty acid ratio and maternal fat content on gonadal fat weight in male (*p* < 0.01) and female (*p* < 0.05) offspring. *n* = 6–9 per dietary group.

**Table 2:** Offspring lipid and liver characteristics at 4 and 8 weeks of age.

Experimental Group	Male				Female			
	High LA (18% Fat)	High LA (36% Fat)	Low LA (18% Fat)	Low LA (36% Fat)	High LA (18% Fat)	High LA (36% Fat)	Low LA (18% Fat)	Low LA (36% Fat)
**4 Week Offspring**								
Plasma cholesterol (mmol/L)	2.93 ± 0.13	3.08 ± 0.10	3.08 ± 0.13	2.90 ± 0.15	2.83 ± 0.09	2.90 ± 0.18	2.80 ± 0.14	2.55 ± 0.10
Plasma TAG (mmol/L)	0.89 ± 0.07 ^a^	0.74 ± 0.03 ^b^	1.03 ± 0.12 ^a^	0.75 ± 0.06 ^b^	0.72 ± 0.06	0.76 ± 0.05	0.83 ± 0.09	0.80 ± 0.09
Liver lipid (mg/g tissue)	30.59 ± 4.37	33.60 ± 3.51	28.55 ± 6.07	33.53 ± 1.28	34.33 ±7.35	36.61 ± 3.25	26.03 ± 3.01	28.57 ± 4.00
Liver cholesterol (mg/g tissue)	1.93 ± 0.20 ^a^	1.46 ± 0.12 ^b^	1.78 ± 0.15 ^a^	1.63 ± 0.11 ^b^	1.63 ± 0.18	1.58 ± 0.12	1.56 ± 0.15	1.55 ± 0.09
Liver TAG (mg/g tissue)	18.01 ± 1.71	16.40 ± 1.54	16.01 ± 2.64	13.23 ± 1.90	17.05 ± 2.42 ^a^	15.75 ± 1.37 ^a^	13.99 ± 1.03 ^b^	11.56 ± 0.85 ^b^
Liver *Fads1* ^#^	0.99 ± 0.15	1.31 ± 0.21	0.88 ± 0.07	0.97 ± 0.20	1.09 ± 0.06	1.48 ± 0.21	1.02 ± 0.10	0.82 ± 0.08
Liver *Fads2* ^#^	1.08 ± 0.13	1.42 ± 0.18	1.27 ± 0.19	1.16 ± 0.16	1.19 ± 0.06	1.54 ± 0.16	1.33 ± 0.19	0.95 ± 0.06
**8 Week Offspring**								
Plasma cholesterol (mmol/L) *	2.39 ± 0.14	3.47 ± 0.33	2.87 ± 0.14	2.97 ± 0.15	2.30 ± 0.30	2.72 ± 0.21	2.72 ± 0.17	2.64 ± 0.15
Plasma TAG (mmol/L)	1.57 ± 0.18	1.48 ± 0.17	1.42 ± 0.09	1.51 ± 0.11	1.10 ± 0.16	0.92 ± 0.06	1.11 ± 0.13	1.03 ± 0.11
Liver lipid (mg/g tissue)	37.56 ± 3.69	36.18 ± 6.82	28.22 ± 10.63	26.27 ± 3.47	26.81 ± 2.27	32.73 ± 6.80	30.99 ± 5.70	37.27 ± 7.01
Liver cholesterol (mg/g tissue)	1.20 ± 0.06	1.06 ± 0.12	1.11 ± 0.12	0.97 ± 0.10	0.87 ± 0.13	1.16 ± 1.38	0.89 ± 0.17	0.94 ± 0.12
Liver TAG (mg/g tissue) *	19.87 ± 1.80	9.81 ± 1.13	13.68 ± 1.99	12.50 ± 1.87	14.53 ± 1.29	12.15 ± 2.53	11.72 ± 0.89	8.91 ± 1.24

All values are mean ± SEM. A two-way ANOVA was used to analyse results with maternal dietary fatty acid ratio and maternal dietary fat content as factors; all comparisons are made within sex group. Different superscripts (a, b) denote values which are significantly different (*p* < 0.05). A significant interaction of maternal dietary fat content and maternal dietary fatty acid is denoted for * males (*p* < 0.05) and ^#^ females (*p* < 0.05). *n* = 6–9 per dietary group.

**Table 3:** Offspring lipogenic gene expression in male and female offspring at 4 weeks of age.

Experimental Group	Male				Female			
	High LA (18% Fat)	High LA (36% Fat)	Low LA (18% Fat)	Low LA (36% Fat)	High LA (18% Fat)	High LA (36% Fat)	Low LA (18% Fat)	Low LA (36% Fat)
**4 Week Offspring**								
Liver								
*Fasn*	0.91 ± 0.12	0.93 ± 0.15	1.49 ± 0.42	0.66 ± 0.08	0.97 ± 0.14	1.08 ± 0.10	1.12 ± 0.19	0.90 ± 0.08
*Lpl* ^†,#^	0.12 ± 0.01	0.13 ± 0.01	0.10 ± 0.01	0.18 ± 0.02	0.15 ± 0.02	0.11 ± 0.01	0.10 ± 0.01	0.18 ± 0.03
*Pparg*	0.95 ± 0.18	0.91 ± 0.11	0.94 ± 0.17	0.91 ± 0.17	0.96 ± 0.22	0.78 ± 0.12	0.70 ± 0.10	0.75 ± 0.13
*Srebf1*	0.82 ± 0.13 ^a^	0.64 ± 0.02 ^b^	0.89 ± 0.11 ^a^	0.52 ± 0.07 ^b^	0.86 ± 0.10 ^a^	0.65 ± 0.07 ^b^	0.79 ± 0.10 ^a^	0.40 ± 0.05 ^b^
Gonadal Fat								
*Fasn* ^#^	3.23 ± 1.17	2.22 ± 0.52	2.36 ± 0.37	1.17 ± 0.06	0.85 ± 0.16	2.42 ± 0.46	3.08 ± 0.47	2.29 ± 0.59
*Lpl*	0.90 ± 0.05	0.94 ± 0.13	1.30 ± 0.33	0.80 ± 0.12	1.04 ± 0.30	0.88 ± 0.04	1.47 ± 0.27	1.07 ± 0.17
*Pparg*	0.84 ± 0.04	0.87 ± 0.08	0.95 ± 0.12	0.86 ± 0.03	0.68 ± 0.14	0.80 ± 0.12	0.81 ± 0.06	0.56 ± 0.09
*Srebf1*	3.12 ± 0.65	3.05 ± 0.50	3.29 ± 0.51	2.21 ± 0.27	3.15 ± 0.64 ^a^	2.23 ± 0.18 ^b^	3.68 ± 0.63 ^a^	2.57 ± 0.45 ^b^
*Lep* ^#^	0.90 ± 0.22	0.83 ± 0.08	1.01 ± 0.12	0.62 ± 0.12	0.38 ± 0.05	0.72 ± 0.12	1.32 ± 0.25	0.70 ± 0.14
Retroperitoneal Fat								
*Fasn*	5.19 ± 1.09 ^a^	2.35 ± 0.37 ^b^	3.01 ± 0.37 ^a^	2.19 ± 0.45 ^b^	1.86 ± 0.42	2.11 ± 0.47	2.13 ± 0.16	1.77 ± 0.57
*Lpl*	1.67 ± 0.16 ^a^	1.13 ± 0.17 ^b^	1.46 ± 0.11 ^a^	1.19 ± 0.18 ^b^	1.21 ± 0.14 ^a^	0.99 ± 0.11 ^b^	1.41 ± 0.13 ^a^	0.86 ± 0.04 ^b^
*Pparg* ^#^	0.72 ± 0.03	0.95 ± 0.09	1.03 ± 0.20	1.15 ± 0.25	0.70 ± 0.07	1.01 ± 0.11	1.12 ± 0.22	0.71 ± 0.05
*Srebf1*	2.71 ± 0.19 ^a^	1.89 ± 0.10 ^b^	2.47 ± 0.33 ^a^	1.74 ± 0.15 ^b^	2.93 ± 0.53 ^a^	2.27 ± 0.17 ^b^	2.61 ± 0.40 ^a^	1.77 ± 0.20 ^b^
*Lep* ^#^	2.23 ± 0.39 ^a^	1.31 ± 0.10 ^b^	1.84 ± 0.15 ^a^	1.43 ± 0.20 ^b^	1.00 ± 0.08	1.03 ± 0.11	1.77 ± 0.13	0.95 ± 0.17

Values are means ± SEM and *n* = 6–9 per group. The effects of maternal dietary fatty acid ratio and maternal dietary fat content were analysed using a two-way ANOVA; all comparisons are made within sex group. Different superscripts (a, b) denote statistical different (*p* < 0.05). A significant interaction of maternal dietary fat content and maternal dietary fatty acid is denoted for ^†^ males (*p* < 0.05) and ^#^ females (*p* < 0.05). *Fasn*, fatty acid synthase; *Lpl*, lipoprotein lipase; *Pparg*, peroxisome proliferator-activated receptor gamma; *Srebf1*, sterol regulatory element-binding protein (variant 1c); *Lep*, leptin.

**Table 4:** Offspring lipogenic gene expression in male and female offspring at 8 weeks of age.

Experimental Group	Male				Female			
	High LA (18% Fat)	High LA (36% Fat)	Low LA (18% Fat)	Low LA (36% Fat)	High LA (18% Fat)	High LA (36% Fat)	Low LA (18% Fat)	Low LA (36% Fat)
Liver								
*Fasn* ^†^	1.40 ± 0.14	2.23 ± 0.5	2.20 ± 0.34	1.58 ± 0.13	1.75 ± 0.16	2.45 ± 0.45	1.59 ± 0.29	2.43 ± 0.40
*Lpl*	0.12 ± 0.01	0.13 ± 0.02	0.12 ± 0.01	0.12 ± 0.01	0.13 ± 0.01	0.12 ± 0.02	0.10 ± 0.01	0.13 ± 0.01
*Pparg*	2.57 ± 0.53	2.25 ± 0.30	2.03 ± 0.22	1.71 ± 0.07	1.43 ±0.43	1.16 ± 0.29	0.85 ±0.15	0.94 ± 0.15
*Srebf1*	1.80 ± 0.21 ^a^	1.63 ± 0.12 ^b^	2.12 ± 0.25 ^a^	1.56 ± 0.07 ^b^	1.39 ± 0.21	1.27 ± 0.14	1.10 ± 0.18	1.20 ± 0.17
Gonadal Fat								
*Fasn*	2.24 ± 0.64	2.70 ± 0.53	2.83 ± 0.51	4.03 ± 0.88	1.22 ± 0.35 ^a^	2.54 ± 0.23 ^b^	2.06 ± 0.59 ^a^	4.36 ± 1.10 ^b^
*Lpl*	2.91 ± 0.31	3.55 ± 0.52	3.50 ± 0.63	3.12 ± 0.36	2.00 ± 0.38	3.65 ± 0.78	3.06 ± 0.43	3.27 ± 0.53
*Pparg*	1.02 ± 0.08	0.90 ± 0.11	0.82 ± 0.06	1.00 ± 0.09	0.92 ± 0.07	0.94 ± 0.12	0.89 ± 0.14	1.02 ± 0.11
*Srebf1*	3.18 ± 0.46	2.98 ± 0.17	3.65 ± 0.47	3.33 ± 0.56	2.48 ± 0.27	2.97 ± 0.33	3.14 ± 0.55	3.81 ± 0.69
*Lep*	4.79 ± 0.76 ^a^	4.12 ± 0.52 ^b^	5.82 ± 0.83 ^a^	3.58 ± 0.44 ^b^	2.34 ± 0.53	2.99 ± 0.47	4.44 ± 0.87	3.31 ± 0.58
Retroperitoneal Fat								
*Fasn*	1.58 ± 0.34	1.85 ± 0.31	1.75 ± 0.17	2.25 ± 0.37	0.64 ± 0.11 ^a^	2.24 ± 0.43 ^b^	1.11 ± 0.36 ^a^	1.53 ± 0.26 ^b^
*Lpl*	2.05 ± 0.45	2.01 ± 0.32	2.01 ± 0.19	2.41 ± 0.42	1.30 ± 0.21	2.08 ± 0.28	1.68 ± 0.21	1.54 ± 0.18
*Pparg*	0.94 ± 0.17	0.92 ± 0.04	0.88 ± 0.05	1.00 ± 0.06	0.92 ± 0.14	1.07 ± 0.09	1.06 ± 0.13	1.01 ± 0.04
*Srebf1*	1.58 ± 0.30	1.48 ± 0.23	1.64 ± 0.10	1.79 ± 0.24	1.06 ± 0.07	1.81 ± 0.24	1.30 ± 0.12	1.29 ± 0.21
*Lep*	2.98 ± 0.68	2.62 ± 0.49	2.85 ± 0.33	2.47 ± 0.47	1.49 ± 0.33	1.95 ± 0.21	1.72 ± 0.24	1.49 ± 0.17

Values are means ± SEM and *n* = 6–9 per group. The effects of maternal dietary fatty acid ratio and maternal dietary fat content were analysed using a two-way ANOVA; all comparisons are made within sex group. Different superscripts (a, b) denote statistical different (*p* < 0.05). ^†^ Indicates a significant interaction of maternal dietary fat content and maternal dietary fatty acid in male offspring (*p* < 0.05). *Fasn*, fatty acid synthase; *Lpl*, lipoprotein lipase; *Pparg*, peroxisome proliferator-activated receptor gamma; *Srebf1*, sterol regulatory element-binding protein (variant 1c); *Lep*, leptin.

## Supplementary Materials

The following are available online at https://www.mdpi.com/2072-6643/12/9/2505/s1, Table S1.
