# Supplementary material for: Omega-6:Omega-3 Fatty Acid Ratio and Total Fat Content of the Maternal Diet Alter Offspring Growth and Fat Deposition in the Rat
Source: Nutrients. 2020 Aug 19;12(9):2505. doi: 10.3390/nu12092505 (PMC7551768; doi:10.3390/nu12092505)
Supplement: Supplementary file 1 [file nutrients-12-02505-s001.pdf]

nutrients

MDPI

Supplementary Table 1. Offspring organ and tissue weights at 4 and 8 weeks of age.

Experimental Group	Male				Female			
	High LA (18% Fat)	High LA (36% Fat)	Low LA (18% Fat)	Low LA (36% Fat)	High LA (18% Fat)	High LA (36% Fat)	Low LA (18% Fat)	Low LA (36% Fat)
**4 Week Offspring**								
Heart (g)	0.54 ± 0.03^{a}	0.48 ± 0.01^{b}	0.54 ± 0.03^{a}	0.41 ± 0.03^{b}	0.51 ± 0.03^{a}	0.44 ± 0.02^{b}	0.51 ± 0.03^{a}	0.41 ± 0.02^{b}
Lungs (g)	1.16 ± 0.04	0.98 ± 0.09	1.09 ± 0.07	1.04 ± 0.07	1.32 ± 0.15^{a}	1.01 ± 0.07^{b}	1.20 ± 0.12^{a}	0.90 ± 0.05^{b}
Kidney (g)	1.21 ± 0.07^{a}	1.03 ± 0.03^{b}	1.18 ± 0.04^{a}	0.86 ± 0.05^{b}	1.12 ± 0.05^{a}	0.89 ± 0.04^{b}	1.12 ± 0.05^{a}	0.81 ± 0.04^{b}
Liver (g)	5.68 ± 0.43^{a}	4.52 ± 0.22^{b}	5.43 ± 0.27^{a}	3.81 ± 0.25^{b}	4.74 ± 0.33^{a}	3.82 ± 0.20^{b}	4.77 ± 0.16^{a}	3.55 ± 0.22^{b}
Gonadal fat (g)*	0.55 ± 0.06	0.47 ± 0.04	0.60 ± 0.04	0.32 ± 0.04	0.59 ± 0.08	0.55 ± 0.07	0.79 ± 0.05	0.42 ± 0.05
Retroperitoneal fat (g)	0.66 ± 0.09^{a}	0.48 ± 0.05^{b}	0.56 ± 0.03^{a}	0.40 ± 0.05^{b}	0.41 ± 0.08^{a}	0.28 ± 0.03^{b}	0.42 ± 0.03^{a}	0.27 ± 0.03^{b}
**8 Week Offspring**								
Brain (g)	1.91 ± 0.05^{a}	1.69 ± 0.05^{b}	1.87 ± 0.02^{a}	1.72 ± 0.03^{b}	1.71 ± 0.05^{a}	1.63 ± 0.05^{b}	1.66 ± 0.04^{a}	1.51 ± 0.04^{b}
Heart (g)	1.17 ± 0.04^{a}	1.09 ± 0.05^{b}	1.23 ± 0.04^{a}	1.14 ± 0.03^{b}	0.83 ± 0.04	0.79 ± 0.03	0.82 ± 0.04	0.76 ± 0.03
Lungs (g)	2.09 ± 0.24	1.83 ± 0.14	2.11 ± 0.19	1.91 ± 0.16	1.15 ± 0.11	1.31 ± 0.06	1.27 ± 0.05	1.28 ± 0.08
Kidney (g)	2.83 ± 0.20^{a}	2.47 ± 0.08^{b}	2.88 ± 0.08^{a}	2.66 ± 0.12^{b}	1.89 ± 0.10	1.70 ± 0.07	1.78 ± 0.06	1.67 ± 0.06
Liver (g)	16.28 ± 0.46^{a}	14.85 ± 0.75^{b}	17.04 ± 0.79^{a}	15.19 ± 0.44^{b}	9.63 ± 0.61	9.06 ± 0.43	9.37 ± 0.35	8.70 ± 0.24
Gonadal fat (g)	4.43 ± 0.40^{a}	3.73 ± 0.31^{b}	4.62 ± 0.38^{a}	3.62 ± 0.32^{b}	2.95 ± 0.32	2.83 ± 0.11	3.47 ± 0.27	3.01 ± 0.46
Retroperitoneal fat (g)	4.11 ± 0.30	3.88 ± 0.41	4.55 ± 0.40	3.52 ± 0.31	1.89 ± 0.26	1.89 ± 0.32	2.01 ± 0.21	1.40 ± 0.05

All values are mean ± SEM. A two-way ANOVA was used to analyse results with maternal dietary fatty acid ratio and maternal dietary fat content as factors, all comparisons are made within sex groups. Different superscripts denote values which are significantly different ($P<0.05$). * indicates a significant interaction effect of maternal dietary fatty acid ratio and maternal fat content on gonadal fat weight in male and female offspring ($P<0.05$). n=6-9 per dietary group.

© 2020 by the authors. Submitted for possible open access publication under the terms and conditions of the Creative Commons Attribution (CC BY) license (http://creativecommons.org/licenses/by/4.0/).

Nutrients **2020**, *12*, 2489; doi:10.3390/nu12082489
